# Temperature and transmural region influence functional measurements in unloaded left ventricular cardiomyocytes

**DOI:** 10.1002/phy2.158

**Published:** 2013-11-14

**Authors:** Charles S Chung, Kenneth S Campbell

**Affiliations:** Department of Physiology and Center for Muscle Biology, University of KentuckyLexington, Kentucky

**Keywords:** Calcium, contraction, relaxation, sarcomere

## Abstract

Intact cardiomyocytes are increasingly being used to investigate the molecular mechanisms of contraction and to screen new therapeutic compounds. The function of the cardiomyocytes is often measured from the calcium transients and sarcomere length profiles. We studied the role of experimental temperature and transmural region on indices of function in freshly isolated, unloaded cardiomyocytes. Intact cardiomyocytes were isolated from the subendocardium, midmyocardium, and subepicardium of 3-month-old Sprague-Dawley rats. Myocytes from each region were studied at 25°C, 31°C, and 37°C. Cytosolic calcium transients were measured using Fura-2 fluorescence, whereas sarcomere length shortening and relengthening profiles were measured using high-speed video capture. For both the calcium transients and sarcomere length profiles, the time to peak and the time to half relaxation decreased significantly with increasing temperature. Increasing temperature also raised the minimum and maximum calcium levels of all cells. Of note, there was a reduced coefficient of variation (standard deviation divided by the mean) at higher temperatures for calcium fluorescence amplitudes, time to peak calcium, and rates of sarcomeric shortening and relengthening. The amplitudes and minimum of the calcium transients were significantly dependent on transmural region, and several sarcomere length parameters exhibited statistical interactions between temperature and transmural region. Together, these results show that biological variability can be reduced by performing experiments at 37°C rather than at room temperature, and by isolating cells from a specific transmural region. Adopting these procedures will improve the statistical power of subsequent analyses and increase the efficiency of future experiments.

## Introduction

Intact cardiomyocytes are increasingly being used to investigate the molecular mechanisms of cardiomyocyte contractility and to screen therapeutic compounds that could be used to treat cardiac diseases (King et al. 2011; Malik and Morgan 2011; Aronson and Krum 2012; Campbell et al. 2013). New compounds could help millions of patients by changing calcium transients or cross-bridge function (Massie et al. 2008; Kitzman et al. 2010; Borlaug and Paulus 2011; Zile et al. 2011; Steinberg et al. 2012). However, the effects of some experimental factors that may influence cardiomyocyte function have not yet been quantified. New data accounting for these factors could be used to improve experimental designs and to increase the efficiency of future work with single cells.

One experimental factor is temperature, which is known to influence both cross-bridge kinetics (Gordon et al. 2000) and calcium (Ca^2+^) transients (Fu et al. 2005). For example, it is well known that the rate of force redevelopment after rapid shortening and restretch (commonly referred to as k_tr_) is faster at higher temperatures (Campbell and Holbrook 2007; Milani-Nejad et al. 2013). Additional data about the effects of temperature on the contractile function of intact tissues have also been reported (Stuyvers et al. 2002), but the temperature dependence of unloaded shortening and relengthening does not appear to have received detailed analysis. Similarly, whereas the temperature dependence of calcium sparks has been studied (Fu et al. 2005), the influence of temperature on calcium transients has not been systematically evaluated in freshly isolated, electrically excitable cardiomyocytes.

Another factor that is not generally considered in cardiomyocyte experiments is transmural variation. Clinically, the function (strain) of the midmyocardial region of the left ventricle is a key predictor of outcomes in patients with heart failure (Vinch et al. 2005; Wachtell et al. 2010). Sarcomeric protein expression and phosphorylation are also known to be dependent on transmural region (Cazorla and Lacampagne 2011; Van Der Velden et al. 2011; Campbell et al. 2013). If these effects lead to significant functional differences, then screening of myocytes may be improved by analyzing cells from defined transmural regions.

## Methods

### Animals

Six female Sprague-Dawley rats (3.0 ± 0.2 months of age; Harlan, Indianapolis, IN) were used in this study. All animal use was approved by the Institutional Animal Use and Care Committee of the University of Kentucky.

### Experiments

Cardiomyocytes were isolated as previously described (Louch et al. 2011; Campbell et al. 2013). Briefly, rats were heparinized (700 U i.p.), anesthetized (Sodium Pentobarbitol 50 mg kg^−1^ i.p.), and then subsequently killed by exsanguination. Excised hearts were immediately placed in a cold (4°C), oxygenated (>10 ppm) perfusion solution (in mmol/L: 113 NaCl, 4.7 KCl, 0.6 KH2PO_4_, 1.2 MgSO_4_, 12 NaHCO_3_, 10 KHCO_3_, 10 4-(2-hydroxyethyl)-1-piperazineethanesulfonic acid [HEPES], 30 Taurine, 5.5 glucose, and 10 2,3-butanedione monoxime [BDM]). The hearts were rapidly cannulated and flushed with 5 mL of cold perfusion solution and then with ∼10 mL warm (37°C, 90 mmHg pressure) perfusion solution until they were completely cleared of blood. The perfusate was then changed to warm (37°C) digestion solution (perfusion solution without BDM, but containing 2 *μ*mol/L CaCl_2_ and 1.4 mg of Liberase TH™ [Roche, Indianapolis, IN]). Hearts were digested until soft and spongy (∼12 min) and then removed from the cannula. The left ventricular free wall was divided into three evenly sized transmural regions that were defined as the subendocardial (endo), midmyocardial (mid), and subepicardial (epi) regions. Separated tissues were minced using small, ceramic-coated scissors, lightly agitated to dissociate cells, and then resuspended with stop solution A (perfusion solution with 10% fetal bovine serum (FBS) and 12 *μ*mol/L CaCl_2_) in individual 2 mL Eppendorf tubes for 5 min. The supernatant was then removed and the cells resuspended in stop solution B (perfusion solution with 5% FBS and 12 *μ*mol/L CaCl_2_) for a further 5 min. Finally, extracellular calcium was raised to 1 mmol/L using a 5-step calcium ladder. The cells were allowed to acclimate for at least 1 h before use. All solutions were pH 7.3.

Each of the three transmural regions was studied for all hearts. Functional measurements were performed in an oxygenated (>10 ppm) Tyrode's solution (in mmol/L: 140 NaCl, 5.4 KCl, 1.8 CaCl_2_, 1 MgCl_2_, 10 HEPES, and 10 glucose). pH was adjusted to 7.3 for separate volumes of solution at 25, 31, and 37°C. Thirty minutes before each region was measured, 40 *μ*L of a solution containing calcium tolerant cells was added to 230 *μ*L of Tyrode's solution with 0.02% pluronic acid and 2 *μ*mol/L of the cell permeable fluorescent calcium indicator Fura-2AM (Life Technologies, Grand Island, NY). Fura was allowed to incorporate into the cytosol for 15 min. A volume of 30 *μ*L of the solution containing calcium tolerant cells was then placed in 150 *μ*L of Tyrode's solution to prevent an excess loading of Fura, or loading into organelles. After 15 min, cells were loaded at 25°C into a custom temperature controlled chamber that was continuously perfused with oxygenated Tyrode's solution. Temperature was controlled using an in-line heater (HPRE2; Cell MicroControls, Norfolk, VA) and a custom 1.5 W heating source formed from power resistors embedded around the chamber. After settling to the cover glass, cells were continuously paced at 0.5 Hz using a 5-msec duration bipolar excitation throughout the experiment. Temperature changes were performed in 3°C steps (∼5 min per step) to allow for replacement of solution at the correct pH.

Measurements were performed using apparatus previously described by Campbell et al. (2013). Briefly, sarcomere length profiles were measured using a high-speed video camera and converted into an analog voltage signal using a D/A converter (901A HVSL and 903A; Aurora Scientific, Aurora, Ontario, Canada). Calcium transients were measured by monitoring Fura-2 fluorescence at 510 nm in response to dual wavelength excitation at 340 and 380 nm (Ratiomaster DeltaRAM X illuminator and D-104 Photometer; Photon Technology International, Birmingham, NY). Excitation switching, temperature, and pacing were computer controlled using a customized program written in MATLAB (Mathworks, Natick, MA) and a 16 bit data acquisition card (DAP5216a; Microstar Laboratories, Bellevue, WA) operating at 2 kHz.

**Figure 1:**
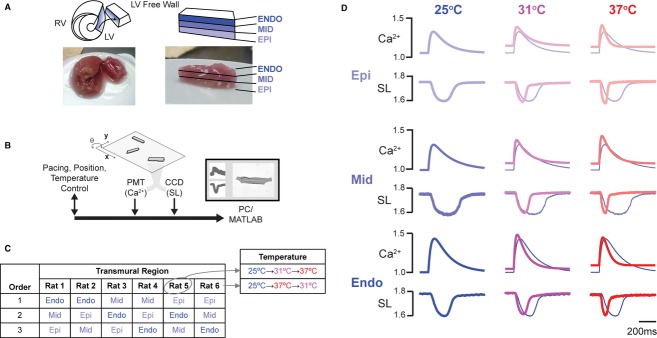
Experiment overview and example data. (A) Schematic (top) and images (bottom) of separation of transmural regions from the LV free wall. After hearts were removed from the cannulae, the LV free wall was dissected (left) and separated into three transmural regions: subepicardium (Epi), midmyocardium (Mid), and subendocardium (Endo) of even thickness (right), dissociated, and myocytes were isolated from each region. (B) System Schematic. Calcium (Ca^2+^) transients and sarcomere length (SL) profiles were measured using a photomultiplier tube (PMT) and a CCD camera, respectively. A computer-controlled system allowed repeated measurements of multiple cells at different temperatures. (C) Balanced experimental design. The three transmural regions were analyzed in different orders each of nine rats to minimize the effect of time from isolation. Temperature order was also altered for alternating rats. For example, cells from Rat 5 were taken from 25°C to 31°C and finally 37°C, whereas cells from Rat 6 were taken from 25°C to 31°C and finally 31°C. (D) Example records. Data recorded at 25°C repeated for each transmural region for comparison (thin line). For clarity, calcium data are plotted as best fits to a five-parameter function (Rice et al. 2008) rather than as raw experimental records.

Data were acquired in a series of trials. During each trial, (1) the computer-controlled stage was moved to focus the microscope on a single cell, (2) the stage position was noted for future use, and (3) signals representing sarcomere length and calcium fluorescence emission were acquired for 10 beats. Once ∼10 cells had been measured, the temperature of the chamber was changed and allowed to stabilize (±0.2°C) in Tyrode's solution at the appropriate pH. The next sequence of trials was then initiated. Whenever possible, measurements were repeated on the same cells by moving the stage to the previously recorded positions (Fig. 1).

To reduce the possibility of misinterpreting effects due to the ordering of the measurements, cells from the three transmural regions were studied in different sequences for each rat. The order of the three temperature conditions was also varied. For consistency and cell viability, all experiments began at 25°C. Figure 1C outlines the balanced design.

Calcium transients and sarcomere length twitch profiles were quantified offline using a custom MATLAB script. Calcium fluorescence ratios were calculated by dividing the emission (510 nm) intensity values recorded during successive excitations by 340 and 380 nm wavelengths. Ratios and sarcomere length transients were filtered using Savitzky–Golay filters. The following parameters were determined from the filtered calcium transients: minimum, maximum, and amplitude of the calcium fluorescence ratios, time from the pacing stimulus pulse to the peak fluorescence, and time from the pacing stimulus pulse to the half-relaxation time. The monoexponential time constant of calcium decline (tau) was determined by a fit of the calcium transient for a duration of 1.5 sec starting at the peak. Calcium fluorescence ratios were then normalized by dividing by the mean of the minimum fluorescence ratios measured at 25°C. Additional calculations showed that the results were not compromised by the Savitzky–Golay filtering; values obtained from curve fits to the Ca^2+^ transients derived from a five-parameter function (Rice et al. 2008) exhibited similar trends (data not shown). The parameters obtained from the filtered sarcomere length profiles were as follows: diastolic and systolic sarcomere length, amplitude of shortening, time from the pacing stimulus pulse to the peak shortening, and time from the pacing stimulus pulse to the half-relaxation time. The normalized maximum rates of shortening and relengthening were calculated by normalizing each data set to the diastolic SL calculated using Newton's difference quotient. Sarcomere length records were corrected for a fixed 15 msec delay introduced by the high-speed video sarcomere length system. Data from individual cardiomyocytes were excluded from further analysis if their sarcomere lengths shortened less than 0.1 *μ*m (<5% shortening), their normalized fluorescence ratio amplitudes were less than 0.15, or any of their time parameters were longer than 1 sec. Data were included from all animals (*N* = 6), and measurements were available for the following cells: subepicardial: *n* = 75 at 25°C, *n* = 21 at 31°C, *n* = 19 at 37°C; midmyocardial: *n* = 64 at 25°C, *n* = 16 at 31°C, *n* = 19 at 37°C; and subendocardial: *n* = 38 at 25°C, *n* = 11 at 31°C, *n* = 12 at 37°C.

Separate sets of cells were used to test whether the observed experimental effects were independent of pH and/or rundown (Fig. 1C). One set was measured 25°C over 1.5 h to evaluate rundown. A second set was used to evaluate if pH errors would alter our measurements. Our solutions were set to a pH of 7.3 as it is the normal physiologic pH. Increasing the temperature of Tyrode's solution from 25°C to 37°C caused the pH to drop to 7.0; therefore, all solutions were set to the correct pH in three separate volumes. To evaluate if pH influenced our results, we measured cells at 25°C in solutions where pH was set to 7.3 and then retested in solutions where pH was set to 7.0 (the pH of an uncorrected solution at 37°C). Measurements were performed as described above.

### Statistics

Statistical analysis was performed using SAS (9.3, SAS Institute, Cary, NC). The experimental data were analyzed using linear mixed models with temperature and region as the main effects and individual cells as the random grouping variable. This approach maximizes the statistical power when multiple cells can be repeatedly recorded from each transmural region. The main effects of the linear mixed model describe the behavior of all cells within that factor alone (either temperature or transmural region). The behavior of cells when subdivided by both temperature and transmural region (interaction) was also considered. Calculations were implemented using the GLIMMIX procedure in SAS assuming compound symmetry for the covariance structure. Post hoc tests were performed using Tukey's multiple comparison tests to determine significance between specific conditions. Data were reported as mean ± SD; a *P*-value less than 0.05 was considered significant.

## Results

### Dependence of calcium transients on temperature and region

**Figure 2:**
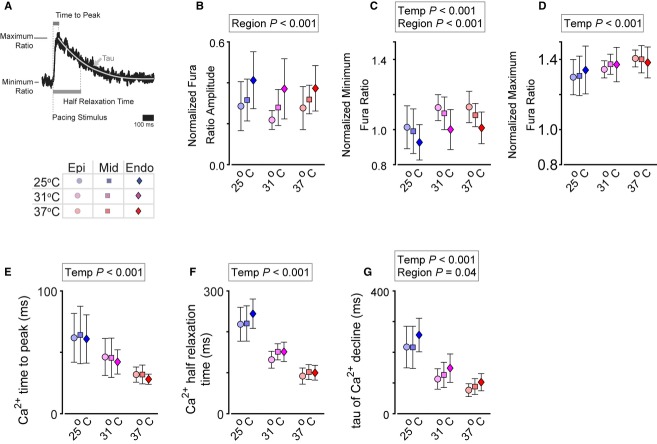
Effect of temperature and transmural region on calcium transients. (A) A raw calcium transient labeled to show calculated parameters. Panels (B–D) show the normalized amplitude, minimum, and maximum of the calcium transients, respectively. Panels (E–G) show time from pacing to peak fluorescence, half-relaxation time, and tau of the calcium decline, respectively. Symbols show Mean ± SD. Statistically significant main effects are shown in the inset boxes. See Table 1 for complete parameters and post hoc tests.

Calcium transients, derived from normalized Fura-2 fluorescence ratios, were dependent on temperature and the transmural region from which the cells were isolated (Fig. 2, Table 1). The minimum fluorescence ratio was temperature dependent, with higher fluorescence at higher temperatures (Fig. 2C). This was paralleled by a similar temperature dependence in the maximum fluorescence ratio (Fig. 2D). Thus, the amplitude of the ratio change (maximum minus minimum amplitude) was not temperature dependent (Fig. 2B). However, the fluorescence amplitude showed a transmural dependence with cells from the subendocardial region exhibiting the largest transients. This reflects an effect of region on the minimum (diastolic) fluorescence ratio. Cells from the subendocardial region had lower minimum fluorescence ratios than cells from the other regions isolated (Fig. 2C, Table 1).

**Table 1:** Measured properties of calcium transients

*Normalized fura ratio amplitude*						
Temp: *P* = 0.33		Region: *P* < 0.001		Temp × Region: *P* = 0.97		

	25°C		31°C		37°C	
Subepicardial	0.281 ± 0.118		0.218 ± 0.046		0.277 ± 0.105	
Midmyocardial	0.313 ± 0.103		0.275 ± 0.087		0.320 ± 0.068	
Subendocardial	0.391 ± 0.137	<0.001 vs. Epi, <0.001 vs. Mid	0.370 ± 0.147	<0.001 vs. Epi, <0.01 vs. Mid	0.373 ± 0.111	<0.01 vs. Epi, <0.05 vs. Mid

*Normalized minimum fura ratio*						
Temp: *P* < 0.001		Region: *P* < 0.001		Temp × Region: *P* = 0.67		

	25°C		31°C		37°C	
Subepicardial	1.022 ± 0.125		1.125 ± 0.074	<0.01 vs. 25	1.129 ± 0.090	<0.001 vs. 25°C
Midmyocardial	1.005 ± 0.127		1.098 ± 0.092	<0.05 vs. 25	1.080 ± 0.065	<0.05 vs. 25°C
Subendocardial	0.948 ± 0.110	<0.01 vs. Epi, <0.05 vs. Mid	1.001 ± 0.114	<0.05 vs. Epi	1.010 ± 0.090	<0.01 vs. Epi

*Normalized maximum fura ratio*						
Temp: *P* < 0.001		Region: P = 0.58		Temp × Region: *P* = 0.66		

	25°C		31°C		37°C	
Subepicardial	1.303 ± 0.099		1.344 ± 0.049		1.405 ± 0.048	<0.001 vs. 25°C, <0.05 vs. 31°C
Midmyocardial	1.318 ± 0.112		1.372 ± 0.055		1.400 ± 0.076	<0.05 vs. 25°C
Subendocardial	1.339 ± 0.127		1.371 ± 0.098		1.382 ± 0.088	

*Calcium time to peak (msec)*						
Temp: *P* < 0.001		Region: *P* = 0.65		Temp × Region: *P* = 0.95		

	25°C		31°C		37°C	
Subepicardial	61.4 ± 20.3		46.1 ± 15.1	<0.01 vs. 25°C	31.8 ± 6.2	<0.001 vs. 25°C
Midmyocardial	64.7 ± 25.6		47.6 ± 17.6	<0.01 vs. 25°C	31.8 ± 7.4	<0.001 vs. 25°C
Subendocardial	62.3 ± 23.1		42.2 ± 9.9	<0.01 vs. 25°C	28.0 ± 4.2	<0.001 vs. 25°C

*Calcium half relaxation time (msec)*						
Temp: *P* < 0.001		Region: *P* = 0.19		Temp × Region: *P* = 0.19		

	25°C		31°C		37°C	
Subepicardial	215.8 ± 43.8		131.8 ± 20.6	<0.001 vs. 25°C	91.7 ± 19.7	<0.001 vs. 25°C, <0.001 vs. 31°C
Midmyocardial	215.1 ± 46.0		149.5 ± 20.2	<0.001 vs. 25°C	102.1 ± 17.8	<0.001 vs. 25°C, <0.001 vs. 31°C
Subendocardial	241.2 ± 34.3		151.0 ± 23.8	<0.001 vs. 25°C	99.9 ± 18.1	<0.001 vs. 25°C, <0.001 vs. 31°C

*Tau of the Calcium decline (msec)*						
Temp: *P* < 0.001		Region: *P* = 0.040		Temp × Region: *P* = 0.78		

	25°C		31°C		37°C	
Subepicardial	214.2 ± 67.3		112.9 ± 33.3	<0.001 vs. 25°C	76.7 ± 20.9	<0.001 vs. 25°C
Midmyocardial	207.9 ± 71.3		121.6 ± 43.7	<0.001 vs. 25°C	89.3 ± 25.5	<0.001 vs. 25°C
Subendocardial	245.1 ± 59.0	<0.01 vs. Epi, <0.05 vs. Mid	148.2 ± 46.4	<0.001 vs. 25°C	102.4 ± 27.9	<0.001 vs. 25°C

For each measured property, main statistical effects are listed followed by data shown as mean ± SD. Significant *P*-values from Tukey's adjusted multiple comparisons tests are also listed.

The timing of the transients was strongly dependent on temperature, but exhibited few region-specific effects. The time to peak and time to half relaxation of the calcium transient were temperature dependent (Fig. 2E and F, Table 1). Time to peak values became shorter as temperature increased from 25°C to 31°C, and underwent further (but nonsignificant) reductions between 31°C and 37°C. Half-relaxation times measured at the three temperatures were significantly different from each other. Neither of these parameters was dependent on transmural region (Fig. 2E and F, Table 1). The time constant of calcium reuptake (tau) indicates how long it takes to remove calcium from the cytosol. This parameter not only showed a dependence on temperature but also exhibited regional dependence (Figs. 2G and 5, Table 1). There were no significant interactions (temperature × region) (Table 1).

The standard deviation for all timing measurements fell as the temperature increased. In part, this reflects the effect of temperature on the mean value of the measurements. However, the coefficient of variation (standard deviation divided by the mean) decreased with temperature for the minimum and maximum fluorescence ratios and the time to peak of this signal. Measurements of fluorescent signals at 37°C thus have proportionately less experimental variability than measurements performed at lower temperatures. There were no clear region-dependent changes in variance.

### Dependence of sarcomere twitch on temperature and region

**Figure 3:**
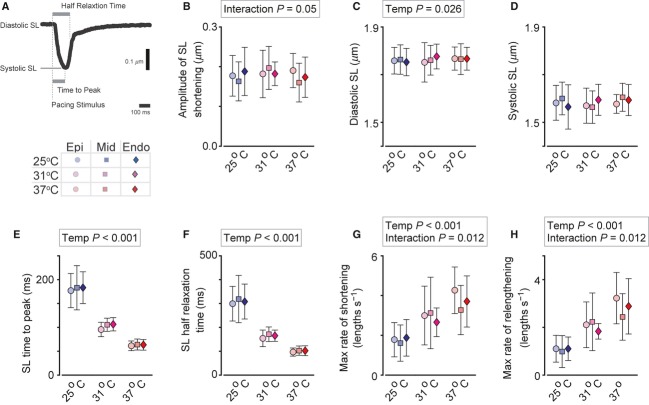
Effect of temperature and transmural region on sarcomere length profiles. (A) A raw sarcomere length profile labeled to show calculated parameters. Panels (B–D) show the amplitude of sarcomeric shortening, and the diastolic and systolic sarcomere lengths, respectively. Panels (E–H) show time from pacing to peak, half-relaxation time, maximum rate of shortening, and the maximum rate of relengthening, respectively. Symbols show Mean ± SD. Statistically significant main effects are shown in the inset boxes. See Table 2 for complete parameters and post hoc tests.

The only statistically significant effect for the amplitudes of the sarcomere length profiles was an interaction between temperature and region (Fig. 3). This implies that the three regions are differentially affected by temperature. The systolic (minimum) sarcomere length was unchanged by temperature and region, but the diastolic sarcomere length was temperature dependent, with slightly higher sarcomere lengths at higher temperatures (Fig. 3C and D, Table 2).

**Table 2:** Measured properties of sarcomere length profiles

*Amplitude of sarcomere length shortening (μm)*						
Temp: *P* = 0.79		Region: *P* = 0.41		Temp × Region: *P* = 0.05		

	25°C		31°C		37°C	
Subepicardial	0.182 ± 0.054		0.182 ± 0.06		0.19 ± 0.043	
Midmyocardial	0.171 ± 0.054		0.197 ± 0.113		0.165 ± 0.053	
Subendocardial	0.19 ± 0.06		0.182 ± 0.143		0.173 ± 0.051	

*Diastolic sarcomere length (μm)*						
Temp *P* = 0.026		Region: *P* = 0.88		Temp × Region: *P* = 0.31		

	25°C		31°C		37°C	
Subepicardial	1.757 ± 0.054		1.751 ± 0.083		1.767 ± 0.048	
Midmyocardial	1.758 ± 0.06		1.757 ± 0.062		1.768 ± 0.064	<0.05 vs. 25°C
Subendocardial	1.753 ± 0.056		1.776 ± 0.052		1.767 ± 0.048	

*Systolic sarcomere length (μm)*						
Temp: *P* = 0.23		Region: *P* = 0.45		Temp × Region: *P* = 0.08		

	25°C		31°C		37°C	
Subepicardial	1.576 ± 0.075		1.57 ± 0.073		1.577 ± 0.039	
Midmyocardial	1.587 ± 0.075		1.561 ± 0.067		1.603 ± 0.058	
Subendocardial	1.563 ± 0.094		1.594 ± 0.065		1.593 ± 0.065	

*Sarcomere length time to peak (systolic length) (msec)*						
Temp: *P* < 0.001		Region: *P* = 0.75		Temp × Region: *P* = 0.98		

	25°C		31°C		37°C	
Subepicardial	174.9 ± 35.7		95.5 ± 15.1	<0.001 vs. 25°C	61.3 ± 10.1	<0.001 vs. 25°C, <0.001 vs. 31°C
Midmyocardial	176.9 ± 46.9		106.2 ± 13.7	<0.001 vs. 25°C	64.2 ± 11.3	<0.001 vs. 25°C, <0.001 vs. 31°C
Subendocardial	181.9 ± 31.2		106.4 ± 14.0	<0.001 vs. 25°C	63.4 ± 11.	<0.001 vs. 25°C, <0.001 vs. 31°C

*Sarcomere length time to half relaxation (msec)*						
Temp: *P* < 0.001		Region: *P* = 0.71		Temp × Region: *P* = 0.99		

	25°C		31°C		37°C	
Subepicardial	294.6 ± 73.0		153.9 ± 34.7	<0.001 vs. 25°C	96.4 ± 17.0	<0.001 vs. 25°C, <0.01 vs. 31°C
Midmyocardial	307.1 ± 98.4		171.7 ± 29.2	<0.001 vs. 25°C	102.4 ± 18.8	<0.001 vs. 25°C, <0.01 vs. 31°C
Subendocardial	303.7 ± 68.7		165.0 ± 23.7	<0.001 vs. 25°C	101.9 ± 20.7	<0.001 vs. 25°C, <0.05 vs. 31°C

*Normalized max rate of shortening* (*length sec*^−1^)						
Temp: *P <* 0.001		Region: *P* = 0.14		Temp × Region: *P* = 0.012		

	25°C		31°C		37°C	
Subepicardial	186.9 ± 92.9		299.4 ± 147.9	<0.001 vs. 25°C	426.7 ± 116.9	<0.001 vs. 25°C, <0.001 vs 31°C
Midmyocardial	182.8 ± 108.2		309.7 ± 174.1	<0.001 vs. 25°C	339.2 ± 131.7	<0.001 vs. 25°C, <0.01 vs. Epi
Subendocardial	194.4 ± 92.9		265.7 ± 72.1		370.3 ± 128.7	<0.001 vs. 25°C, <0.01 vs. 31°C

*Normalized max rate of relengthening (lengths sec*^−1^*)*						
Temp: *P* < 0.001		Region: *P* = 0.17		Temp × Region: *P* = 0.012		

	25°C		31°C		37°C	
Subepicardial	−116.1 ± 58.9		−210.9 ± 95.9	<0.001 vs. 25°C	−322.4 ± 107.6	<0.001 vs. 25°C, <0.001 vs. 31°C
Midmyocardial	−113.2 ± 76.6		−222.7 ± 116.8	<0.001 vs. 25°C	−256.2 ± 109.5	<0.01 vs. Epi
Subendocardial	−113.2 ± 48.7		−183.3 ± 32.9	<0.001 vs. 25°C	−288.3 ± 116.3	<0.001 vs. 25°C, <0.001 vs. 31°C

For each measured property, main statistical effects are listed followed by data shown as mean ± SD. Significant *P*-values from Tukey's adjusted multiple comparisons tests are also listed.

The diastolic sarcomere length, time to peak sarcomere length shortening and the half-relaxation time depended on temperature but not region (Fig. 3E and F, Table 2). However, the diastolic sarcomere length, the amplitude of sarcomere shortening was not dependent on temperature. As the shortening amplitudes were unchanged even though the timing was dramatically altered, the maximum rates of shortening and relengthening were significantly different at all temperatures (Fig. 3G and H, Table 2). A significant statistical interaction between temperature and region was also present in both shortening and relengthening rates, but post hoc tests showed that the specific transmural regions were only significantly different from each other at 37°C (Table 2).

Absolute standard deviations for the time to peak shortening and the time to half relaxation decreased with increasing temperature. Importantly, increasing temperature also decreased the coefficient of variation (SD/mean) for the diastolic and systolic sarcomere lengths, and the rates of shortening and relengthening.

### Control experiments

**Figure 4:**
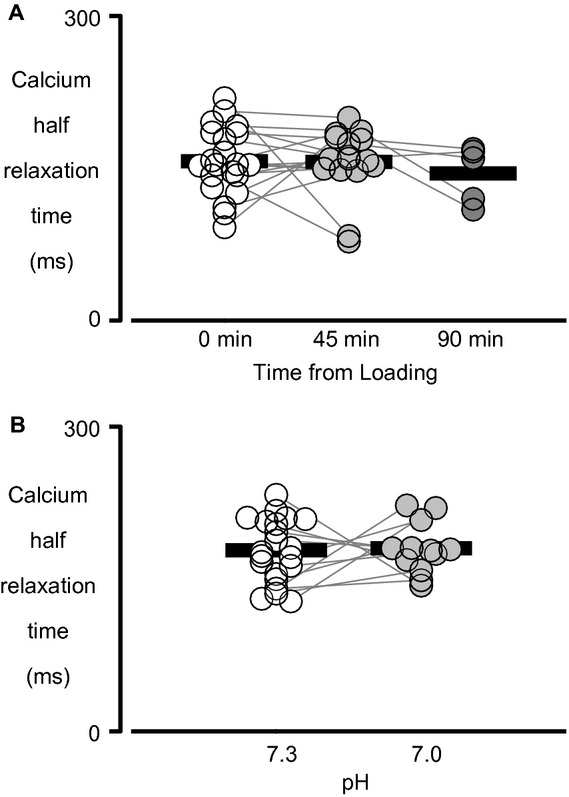
Control experiments. (A) Calcium half-relaxation times measured over 90 min. (B) Calcium half-relaxation times measured at two different pH values. In both panels, lines join repeated measures for the same cell, and thick black bars denote the mean value in each condition.

To determine if the temperature-dependent changes described above were due to the duration of the experiments (∼20 min for each of the three temperatures), 22 cells were measured at three time points over the duration of ∼90 min. While cell death reduced the number of measurements at each time point, there were no significant changes in the mean values of the measured parameters over the course of the experiment (Fig. 4A shows a subset of this data).

Although each experimental solution was set to pH 7.3 at the desired temperature, the influence of pH on parameters was tested to confirm that pH changes did not impact the experimental results. Increasing the temperature of the Tyrode's solution from 25°C to 37°C lowers the pH of the 25°C solution from 7.3 to 7.0. Twenty-two cells were studied (11 repeated measures) beginning at a pH of 7.3 and changing to a pH of 7.0 at 25°C. Eleven cells were lost to cell death or detachment from the cover slide. No change was found that could account for the above-reported results (Fig. 4B shows a subset of this data).

## Discussion

Intact cardiomyocytes can be used to determine both molecular mechanisms of cardiomyocyte function and the effect of potential therapeutic drugs (King et al. 2011; Malik and Morgan 2011; Aronson and Krum 2012). However, the effect of some experimental conditions that could influence the results have not yet been fully quantified. This study shows that, in general, two of these factors – temperature and transmural region – should be accounted for in experiments (Fig. 6).

### Temperature

Although most studies report measurements of cardiomyocytes at 37°C, some publications describe data obtained at room temperature (Griffiths et al. 1997). Studies of cross-bridge kinetics in permeabilized myocardial tissues are sometimes performed at even lower temperatures (e.g., 12°C De Tombe and Stienen 2007). In our experience, experiments with intact myocytes are simpler to perform at room temperature than at 37°C because the cells are viable for longer at the lower temperature. This allows us to measure more cells from a given animal, which in turn improves the statistical power of our analyses. However, we could not find data in the literature that described how the different temperatures would affect our results. The current experiments were designed to help fill this knowledge gap.

The data show that calcium transients became shorter as the experimental temperature is raised to 37°C (Fig. 2, Table 1). This agrees with results from Fu et al. (2005), showing that calcium sparks also become faster at higher temperature. The Q_10_, the change in a parameter corresponding to a 10°C increase in temperature, of the times to peak and half relaxation is ∼0.5, meaning the duration has nearly halved, which is in good correspondence to the normal doubling of rates in biological systems (Reyes et al. 2008). The mechanisms that drive this adaptation are not yet clear and there may be multiple factors at work. For example, calmodulin binding to ryanodine to alter calcium release undergoes large changes in kinetics between 23 and 32°C (Meissner et al. 2009). Sarcoendoplasmic reticulum Ca^2+^/ATPase (SERCA) pumps also clear the cytosolic calcium faster at physiologic temperatures (Liu et al. 1997; Mackiewicz and Lewartowski 2006).

Faster Ca^2+^ transients by themselves would probably lead to shorter duration sarcomere length profiles. However, the Q_10_ of the time to peak and half relaxation for sarcomere lengths is ∼0.4, and the Q_10_ of the rates of shortening and relengthening is 2.5–3.0. These data suggest that the sarcomere length changes cannot be solely dependent on the temperature-dependent doubling in the rate of calcium handling. However, this may be explained by experiments with permeabilized muscle samples that have shown that cross-bridge attachment and detachment rates, and ATPase rates, also increase with temperature (Zhao and Kawai 1994; Campbell and Holbrook 2007; De Tombe and Stienen 2007; Milani-Nejad et al. 2013). Both of these effects probably contribute to the faster sarcomere length transients observed at the higher temperatures used in this work (Fig. 3, Table 2). Temperature may also alter the regulation of contractile activity (Gordon et al. 2000). For example, recent data from murine cells suggest that increasing temperature raises the proportion of myosin heads that can bind to the thin filaments in a low-calcium diastolic state (Chung et al. 2011; King et al. 2011).

Another noteworthy feature of our data was that the coefficient of variation (SD/mean) for some of the experimental parameters decreased with increasing temperature. Specifically, this happened for the maximum and minimum Fura-2 fluorescence ratios, the time to peak Ca^2+^, and the rates of sarcomere length shortening and relengthening. As the coefficient of variation is closely related to an effect size in statistics, this means that hypothesis tests performed with these parameters will have more statistical power at 37°C than they will with data obtained at room temperature. This implies that it may actually be easier to test experimental hypotheses at 37°C, even though cell survival is likely to be more challenging.

With the exception of the amplitudes of the calcium transient and of sarcomere length shortening, temperature is a key factor in the parameters measured from intact cardiomyocytes. Only a few reasons persist in not utilizing physiologic temperature. First, cell survival is improved at lower temperatures which may be important for some experiments. Second, when load control (e.g., work loops) needs to be performed, room-temperature experiments may be simpler to implement because the contractions are slower. However, we recommend that, in general, experiments should be performed at 37°C (Fig. 6).

### Transmural region

Transmural variability is another factor that may influence cell function. Calcium transients have greater amplitudes in cells from the subendocardium than in cells from the subepi- or midmyocardium. This may reflect transmural variation in the expression and phosphorylation of calcium cycling proteins such as SERCA (Laurita et al. 2003; Soltysinska et al. 2009). Transmural variation in SERCA may also explain the slowed subendocardial calcium reuptake (increased tau of the calcium decline). These changes could be clinically relevant given the known transmural differences in calcium transients in normal and failing human hearts (Glukhov et al. 2010; Lou et al. 2011). The regional changes of ∼15% are also a potentially large variant when trying to evaluate if a heart is transitioning to failure or if a treatment improves cardiomyocyte function.

Sarcomere length profiles did not exhibit region-specific effects, but a statistically significant interaction between temperature and transmural region was found for three sarcomere length parameters: shortening amplitude, and the maximal rates of shortening and relengthening (Fig. 3B, G, and H, Table 2). For example, the data reveal that cells from the midmyocardial region have a significantly lower maximum rate of relengthening than cells from the subepicardium at 37°C. In contrast, the rate of relengthening in midmyocardial cells is not lower than that of cells from the subepi- or subendocardium at 31°C. However, there are again trends toward a reduced rate in midmyocardial cells at 25°C. The statistical interaction between temperature and region may indicate that regional differences become masked in subphysiologic temperature experiments. Alternately, physiologic temperatures may enhance transmural differences seen at subphysiologic temperatures (Cazorla and Lacampagne 2011; Van Der Velden et al. 2011; Campbell et al. 2013).

Transmural region may have broader functional implications in the intact heart. A recent study from our lab showed that regional differences may contribute to age-dependent diastolic dysfunction (Campbell et al. 2013). Specifically, the study showed that age-dependent transmural function was associated with changes in torsion. Clinical studies also show that midmyocardial shortening predicts cardiovascular death better than traditional global measures, such as ejection fraction (Vinch et al. 2005; Wachtell et al. 2010). This suggests that organ-level dysfunction may actually be caused by regional effects.

The consequences of transmural changes are potentially large. For example, transmural function is heterogeneous (Vinch et al. 2005; Wachtell et al. 2010; Cazorla and Lacampagne 2011) and the influence of therapeutic agents may augment (or attenuate) the differences between the subepi- and subendocardial layers. Thus, identifying and evaluating how normal intact cardiomyocytes function across transmural layers, and how diseases and therapeutics alter this transmural relationship, could lead to new insights into cardiac function and dysfunction.

**Figure 5:**
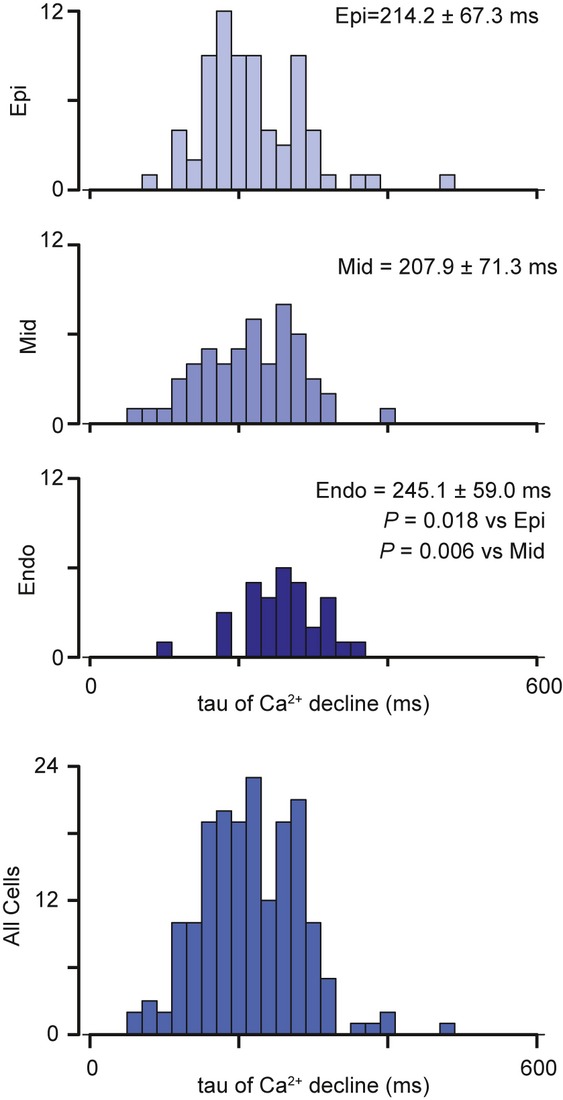
Functional parameters exhibit transmural variation. (A) Histograms showing the number of cells from each myocardial region with given tau values measured at 25ºC. Labels list the Mean ± SD. (B) Histogram showing all data on a single plot.

**Figure 6:**
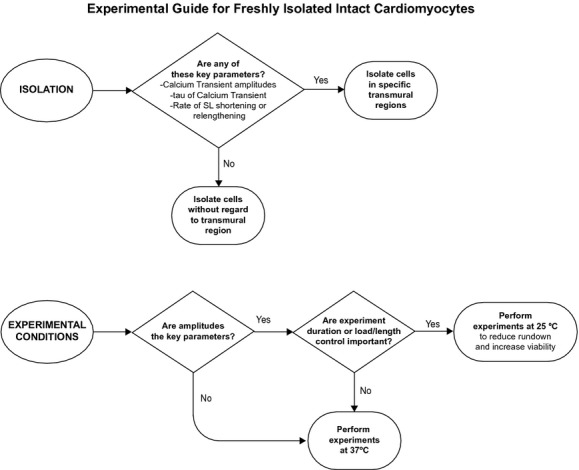
Decision tree to determine optimal experimental conditions.

Whereas some specific parameters (time to peak, etc.) are not dependent on region, parameters including the amplitude and the minimum value of the calcium transient (Fig 2), and the tau of the calcium decline depend on the transmural region from which a cardiomyocyte was isolated (Fig. 5). Thus, restricting experiments to cells isolated from a specific transmural region may reduce biological variability and improve the efficiency of the experimental design (Fig. 6).

### Limitations

A limitation to this study (and most studies of unloaded cardiomyocytes) is the potential disconnect with in vivo function. For example, sarcomere shortening is greater in the subendocardium than in the subepicardium of the intact heart. Diastolic sarcomere lengths are also thought to be >2.0 *μ*m in an intact heart (Streeter and Hanna 1973; Chung and Granzier 2011) compared to the ∼1.75 *μ*m observed in this study. This diastolic length difference reflects the intraventricular pressure (preload) and the extracellular matrix. Besides preload-dependent sarcomere lengths, an unloaded cardiomyocyte does not mimic the in vivo afterload condition where some nearly isometric contraction and relaxation occur.

This study only evaluated freshly isolated intact cardiomyocytes. Investigators are also using cultured or stem cell–derived myocytes. Myocytes from these sources may exhibit different temperature and transmural effects. They also have their own limitations such as myofibrilar disarray (Ehler and Perriard 2000; Diaz and Wilson 2006).

### Summary

This study demonstrates the importance of two experimental factors. First, the amplitude and timing of calcium transients and sarcomere length profiles in unloaded intact cardiomyocytes depend on the experimental temperature. Second, the amplitude and kinetics of calcium transients, and the rates of sarcomere length shortening and relengthening, depend on the transmural region a cell was isolated from. These two experimental parameters should be considered when designing experiments using isolated cardiomyocytes.
